# Characterization of short- and long-term mechanical sensitisation following surgical tail amputation in pigs

**DOI:** 10.1038/s41598-017-05404-y

**Published:** 2017-07-06

**Authors:** Pierpaolo Di Giminiani, Sandra A. Edwards, Emma M. Malcolm, Matthew C. Leach, Mette S. Herskin, Dale A. Sandercock

**Affiliations:** 1 School of Agriculture, Food and Rural Development, Newcastle University, Newcastle upon Tyne, NE1 7RU United Kingdom; 2 Animal and Veterinary Science Research Group, Scotland’s Rural College (SRUC), West Mains Road, Edinburgh, EH16 4SA United Kingdom; 3 Aarhus University, Department of Animal Science, Au-Foulum, Tjele, Denmark

## Abstract

Commercial pigs are frequently exposed to tail mutilations in the form of preventive husbandry procedures (tail docking) or as a result of abnormal behaviour (tail biting). Although tissue and nerve injuries are well-described causes of pain hypersensitivity in humans and in rodent animal models, there is no information on the changes in local pain sensitivity induced by tail injuries in pigs. To determine the temporal profile of sensitisation, pigs were exposed to surgical tail resections and mechanical nociceptive thresholds (MNT) were measured in the acute (one week post-operatively) and in the long-term (either eight or sixteen weeks post-surgery) phase of recovery. The influence of the degree of amputation on MNTs was also evaluated by comparing three different tail-resection treatments (*intact, ‘short tail’, ‘long tail’*). A significant reduction in MNTs one week following surgery suggests the occurrence of acute sensitisation. Long-term hypersensitivity was also observed in tail-resected pigs at either two or four months following surgery. Tail amputation in pigs appears to evoke acute and sustained changes in peripheral mechanical sensitivity, which resemble features of neuropathic pain reported in humans and other species and provides new information on implications for the welfare of animals subjected to this type of injury.

## Introduction

Tissue and nerve injuries caused by surgical procedures lead to acute peripheral sensitisation of the affected area, with a possible involvement of central pain-processing pathways. Animal models of post-incisional pain have for a long time provided a valuable platform to understand the mechanisms mediating alterations in nociceptive thresholds observed shortly after surgical incision or for more sustained periods of time (e.g. days) afterwards^1–4^. Recently, post-operative pain has been investigated in pigs^5^, reporting significant sensitisation to mechanical noxious stimuli (i.e. von Frey stimulation) induced by skin and muscle incision in the caudal end of the lower back. It is well recognised that in situations where there is resection of peripheral nerves the formation of traumatic neuromas (a tangle of truncated neural fibres and connective tissue) can occur, which in humans can be a significant cause of pain^6, 7^. Traumatic neuromas are particularly common in the case of amputation or surgical removal of body parts such as limbs, breasts, rectum, penis, testicles, eye, tongue or teeth^8^. This type of trauma can be associated with the phenomenon of residual stump pain and phantom limb pain, a physiological and psychological percept classified as a neuropathic pain state^8^, with painful symptomatic neuromas observed in 10–25% of amputees^9^.

Neuropathic pain refers to ‘pain arising as a direct consequence of a lesion or disease affecting the somatosensory system^10^, and its physiological manifestation has been investigated in rodent models; however it is yet unknown if a similar phenomenon occurs in pigs following tail amputation and this is an important question in the current debate on animal welfare issues. The aetiology of this particular type of neuropathic pain is uncertain; however persistent ectopic discharge of axons within the neuroma has been suggested as the most plausible mechanism^11^.

Following nerve resection or injury, regenerative sprouting of injured axons occurs, leading to the formation of enlarged and disorganised bundles of nerve endings containing C fibres and demyelinated A fibres exhibiting increased rates and patterns of spontaneous axonal discharge activity^12^. In addition to its effects on the damage and reorganization of peripheral nerve endings, deep tissue injury can cause alterations in peripheral and spinal dorsal horn neuron activity, which can retrogradely affect the function of peripheral sensory afferent nerves (e.g. modulate excitability) and/or feed forward ascendingly to supraspinal centres^13^. This altered activity in spinal and supraspinal regions of the pain processing pathways represents a potential source of sustained pain, such as phantom limb pain experienced by amputees^14^.

In humans, both the peripheral and the central component of the nervous system are believed to be involved in stump pain phenomena^15^, which clinically results in the reduction of pain thresholds (hyperalgesia)^16^. Previous investigations in animals have identified the formation of neuromas following tail amputation in dogs^17^, lambs^18^ and pigs^19–21^. Currently, data on long-term behavioural and physiological consequences of the effect of tail amputation in non-neonatal subjects are lacking in these particular species, however evidence of thermal and mechanical hyperalgesia following tail amputation has been reported in non-neonatal mice^22, 23^. In addition, potentiation of short-term (ca. two weeks) neuronal responses in the somatosensory cortex has been reported in adult rats subjected to third hind paw digit amputation^24, 25^.

Tail amputation is an event frequently observed in pig production as the result of tail docking (a husbandry procedure widely performed on neonatal pigs) or tail biting (an abnormal behaviour that can develop in juvenile pigs). Due to the traumatic nature of the injury, it is highly relevant to investigate its short- as well as long-term consequences on the pain sensitivity of the animals, in order to provide novel information on the underlying mechanisms and to appraise the associated welfare impairment.

Here we describe acute and long-term changes in mechanical nociceptive thresholds induced by a surgical model of tail amputation in pigs, mimicking the injurious tail biting which can occur in both recently weaned and in older growing pigs. We evaluate the influence of (1) age at the time of the surgery; (2) time post-surgery and (3) portion of tail resected.

## Results

A total of 697 single mechanical stimuli were successfully applied as part of the experiment. Eight stimuli (1.1%) did not elicit a clear withdrawal response before reaching the maximal threshold of 1500 gF and were not specifically associated with a particular animal, age or tail treatment (data not shown).

Individual MNT variability, assessed with intra-individual CVs, was on average 28.9% across the entire experimental population. No significant effect of age at time of surgery and tail treatment was detected on CV one week post-surgery (P = 0.679). Similarly, individual variability was not influenced by age at surgery and tail-resection treatment for measurements collected at 8 and 16 weeks post-surgery (P = 0.687 and P = 0.774 respectively).

**Figure 1:**
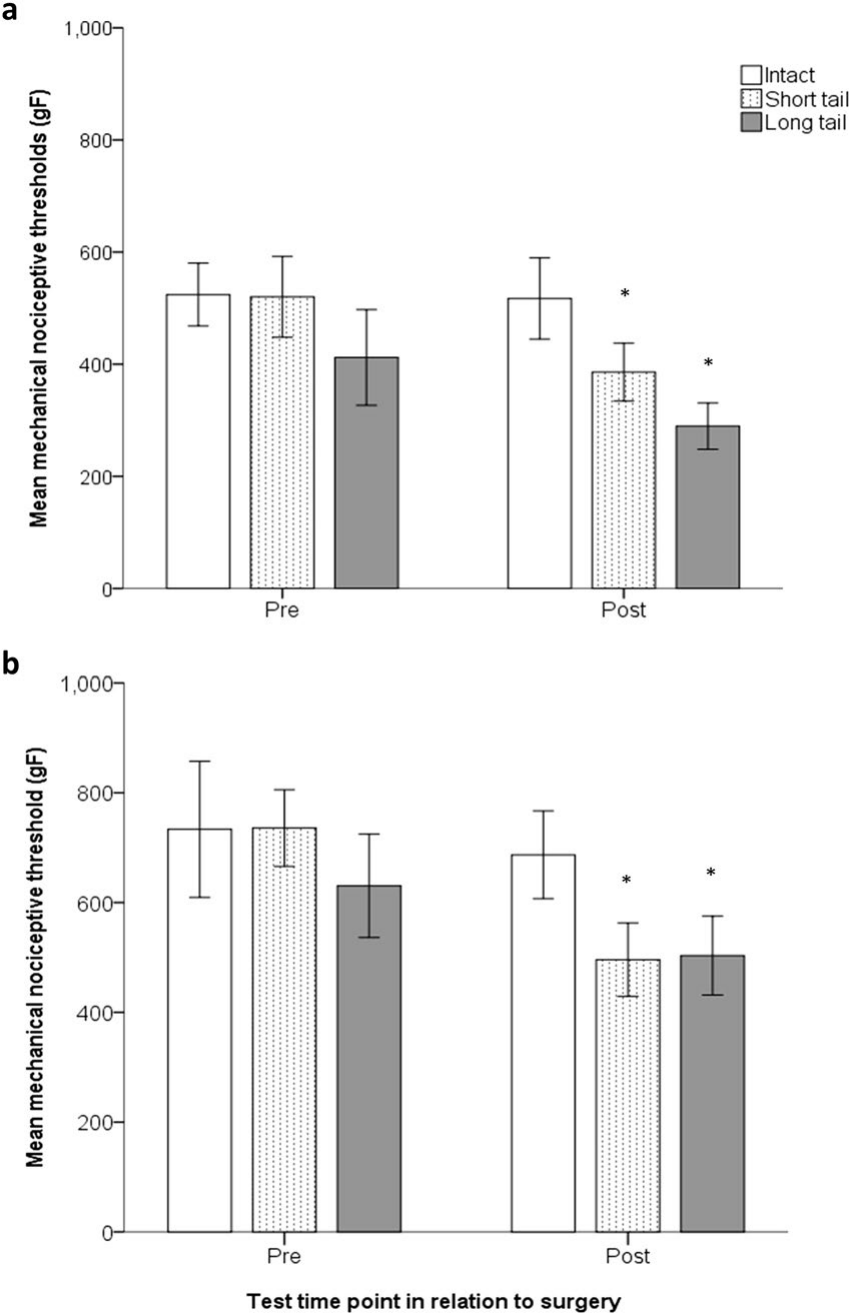
Acute sensitisation one week post-surgery. Mechanical nociceptive thresholds (mean ± SEM) recorded at the site of tail resection 3 days pre- and 1 week post-surgery in pigs undergoing sham/tail amputation surgery (Sham surgery = intact; 2/3rds removed = short tail; 1/3rd removed = long tail) at (**a**) 9 and (**b**) 17 weeks of age. Asterisks indicate significant difference (P < 0.05) between MNTs collected pre- vs. post-surgery.

All pigs with surgically amputated tails exhibited reduced MNTs one week following surgery irrespective of age and tail-resection treatment. MNTs in pigs undergoing surgery at 9 weeks of age (Fig. 1a) significantly decreased for short (pre: 520.2 ± 36.1 gF vs. post: 385.9 ± 25.9 gF; n = 16; P = 0.007) and long tail (pre: 412.1 ± 42.7 gF vs. post: 289.6 ± 20.7 gF; n = 12; P = 0.019) resection groups. MNTs for pigs in the intact tail group did not change following sham surgery (pre: 524.1 ± 36.9 gF vs. post: 517.4 ± 29.2 gF; n = 13; P = 0.869). Similarly, pigs exposed to the surgery at 17 weeks of age (Fig. 1b) had significantly decreased MNTs in the short (pre: 735.8 ± 34.9 gF vs. post: 495.8 ± 33.5 gF; n = 24; P = 0.007) and long tail (pre: 630.5 ± 47.1 gF vs. post: 503.4 ± 35.9 gF; n = 20; P = 0.048) resection groups, whereas no change was recorded in intact tail group (pre: 733.4 ± 62.1 gF vs. post: 686.9 ± 39.9 gF; n = 23; P = 0.490).

**Figure 2:**
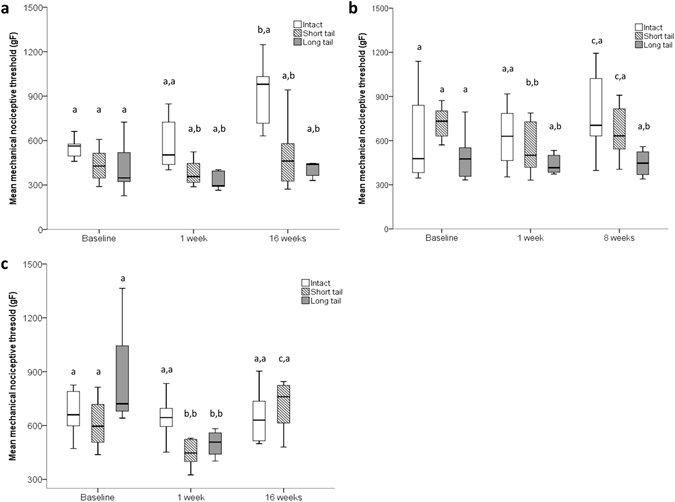
Temporal profile of sensitisation following surgery. Time course of changes in mean mechanical nociceptive thresholds (±SEM) recorded at the site of tail resection across three tail-resection treatments. (**a**) Surgery performed in pigs of 9 weeks of age and MNTs assessed 3 days pre-, 1 week and 16 weeks post-surgery; (**b**) Surgery performed in pigs of 17 weeks of age and MNTs assessed 3 days pre-, 1 week and 8 weeks post-surgery; (**c**) Surgery performed in pigs of 17 weeks of age and MNTs assessed 3 days pre-, 1 week and 16 weeks post-surgery. The vertical bars denote the 25th to 75^th^ and the whiskers 5th to 95^th^ percentile ranges. Different first letters indicate significant difference vs. the previous time point; different second letters indicate significant difference between tail-resection treatment within the same time point (P < 0.05).

After controlling for the effect of pre-surgery MNT, a significant effect of age at surgery (P = 0.003), tail-resection treatment (P < 0.001) and time post-surgery (P = 0.001) was observed. Similarly, a significant interaction of age at surgery and tail-resection treatment (P = 0.001) and a three-way interaction of age at surgery, tail-resection treatment and time post-surgery (P = 0.031) was found (Fig. 2). Significantly lower MNT values were recorded 16 weeks post-tail resection in pigs exposed to surgery at 9 weeks of age when compared to MNTs of intact tails (P < 0.001 for both intact vs. short and long tail comparisons). In pigs undergoing tail resection at 17 weeks of age, MNTs were significantly lower at 8 weeks post-surgery in the long tail treatment (P = 0.001), but did not differ significantly from intact values in the short tail treatment (P = 0.142). At 16 weeks post-surgery no difference in MNTs was recorded between long and intact tail treatments (P = 0.210).

One week following tail amputation MNT values recorded from short and long tail treatments did not differ significantly in animals exposed to surgery at either 9 or 17 weeks of age (P = 0.083 and P = 0.907 respectively). An effect of length of tail resection on MNTs was not observed in animals exposed to surgery at 9 weeks of age when tested 16 weeks post-resection (P = 0.438). In contrast, a significant difference in values of MNT was recorded in pigs exposed to surgery at 17 weeks of age and tested 8 weeks post-resection (P = 0.013).

MNT recording at 8 weeks post-tail resection from pigs exposed to surgery at 9 weeks of age was not possible. This was due to the failure of the habituation protocol to reduce stress associated with the confinement in younger animals, which resulted in frequent escape attempt behaviours and lack of responsiveness to the mechanical stimuli. In order to avoid this occurrence in subsequent groups, the authors opted for the alternative setup with reduced confinement. Due to a technical failure of the PAM device, it was not possible to collect data from the long tail resected pigs 4 months following surgery, therefore the effect of tail amputation length could not be evaluated in this cohort.

## Discussion

The main purpose of this study was to measure the short-and long-term effects of two lengths of tail resection on post-amputation tail stump sensitivity in pigs subjected to surgical tail amputation at two different ages (9 and 17 weeks). The rationale for the study was to investigate the likely effects of such injury in the context of the issue of tail mutilations in pig production, where a proportion of the tail can be fully bitten off, but using an experimental and ethically-approved approach to assess the effects of tail-amputation injury. The ages at which the tail resections were performed also links to the age at which the on-set of tail biting typically occurs in weaner and grower pigs^26^. However, the study also has more general scientific relevance for the fundamental understanding of pain response mechanisms evoked by damages to the peripheral pain pathways.

Data from the present study clearly show that tail amputation at both ages induced short-term changes in mechanical nociceptive thresholds (MNT) representative of an increased sensitivity to noxious mechanical stimulation that appeared to be sustained for a period of up to 16 weeks (4 months) and possibly beyond. MNT data were obtained using a handheld PAM device in conjunction with previously validated assessment and data analysis protocols^27, 28^, through which the consistency of individual levels of MNT, and the variability associated with different animal ages, and anatomical tail locations were determined.

This is the first study to report evidence of immediate and sustained mechanical hyperalgesia following tail amputation injury caused in later life. In addition, the findings of the present study are the first to show that the age or stage of development at which tail amputation injury occurs can affect threshold response profiles, suggesting that 9 week-old animals exposed to the injury experience mechanical sensitisation for a longer period of time than 17 week-old animals, regardless of the extent of tail removed. It is also evident from the results of this study that the amount of tail that is amputated can have a significant effect on the thresholds of tail sensitivity, notably the removal of 1/3 (long tail resection group) of the tail resulting in lower MNT compared with pigs with 2/3 of the tail amputated (short tail resection group), depending on the age of exposure to the surgery.

Similar to our previous observations on pigs with intact tails (97.2%)^27^, a high level of responsiveness to individual stimuli was recorded in this study (98.9%). In addition, the average individual variability of 28.9% observed in the present study corresponds to that reported in our previous experiment (30–32%)^27^, and confirms the efficacy of the habituation procedure on obtaining reliable MNT data. The presence of mechanical hyperalgesia one week after tail resection demonstrated in the present study is consistent with reports of measures of increased sensitisation reported in previous experiments specifically targeting tail amputations or more general tissue injuries. Increased sensitivity to mechanical stimulation following surgical incision of the tail or the plantar surface of the paw can be sustained for a period of up to seven days post-surgery in rodent inflammatory pain models^29, 30^. Similar findings have also been observed in pigs subjected to full skin and muscle incision performed in the lower back^5^. The impact of tail tip removal on mice for DNA profiling has also been previously described in two related studies. Zhuo^22^ and Kim and Zhuo^23^ characterised the development of mechanical and thermal hyperalgesia measured on the dorsal surface of the adult mouse tail, in proximity to the site of injury. Once again, tail sensitisation was sustained for a period of seven days following surgery. None of the previous investigations provided information on MNT profiles extending beyond 7 days post-injury.

It is well recognised that the amputation of limbs and appendages may cause altered sensitivity and possible long-term pain in the affected body part^31^. These effects can be observed in addition to the activation of nociceptive responses at the time of injury and the heightened pain sensitivity that occurs in response to tissue injury and inflammation (i.e. inflammatory pain). Neuropathic pain can arise from injury that occurs to primary sensory neurons responsible for transmitting pain signals, especially when these nerves are resected and subsequently form into neuromas^32^. In the present study, prolonged increases in tail sensitivity were observed up to 4 months after tail amputation in pigs undergoing tail resection at 9 weeks of age. Similarly, enduring sensitivity to noxious mechanical stimulation up to 2 months after injury was also seen in pigs subjected to tail resection at 17 weeks of age, suggesting in both treatment groups that sustained peripheral and spinal somatosensory changes linked to amputation injury are still evident sometime after post-injury tissue inflammation appears to have resolved.

The formation of traumatic neuromas in the stump of tail-docked piglets has been recently described^20, 21^. Both investigations confirmed the presence of traumatic neuromas and neuromatous tissue development in tail stumps collected from pigs of up to 22 weeks of age and suggested that neuroma formation is still incomplete four months after tail docking injury, with possible implications for sensitivity of the tail stump. MNT were not measured in these studies and therefore it was not possible to confirm if the presence or stage of neuroma development had an effect upon post-docking tail stump sensitivity, although it has been previously suggested that the presence of traumatic neuromas may lead to increased sensitivity to pain in the amputation stump^19^.

Amputation-induced injury to the peripheral nerve system, producing neuroanatomical changes such as the development of traumatic neuromas, can be associated with changes in somatosensory nerve function and with consequent alterations in nociceptive afferent fibre activity following nerve transection. These are important factors leading to the experience of abnormal sensations in humans, such as paraesthesia and dysesthesia, and in extreme cases lead to the phenomenon of stump or phantom limb pain^8^. Neuromas have been described as predominantly susceptible to mechanical pressure^33–35^. In addition to showing increased sensitivity to mechanical force, it has been reported in electrophysiological studies on rodents that peripheral nerve traumatic neuromas can exhibit ectopic or spontaneous activity that is linked to mechanical hyperalgesia mediated by damaged and non-damaged A-delta and C-fibre primary afferent neurons^12^. Evidence of sustained reductions in MNT reported in the current study suggests that tail amputation (irrespective of the amount removed) can cause prolonged increases in tail stump sensitivity reflecting mechanical hyperalgesia. This would indicate that tail amputation causes alterations in peripheral and spinal nociceptive processing that lead to central sensitisation^36^ that are still present 2–4 months later and, although the measured responses to mechanical pressure reflect a polysynaptic withdrawal reflex to noxious stimulation, it is reasonable to suggest that the pigs with amputated tails would perceive such mechanical stimulation as more painful (pain hypersensitivity) than their intact counterparts during this time. It is also plausible that the observed mechanical hyperalgesia in pigs subjected to tail amputation in later life may be functionally-linked to the presence of traumatic neuromas in the resected tail stumps, although no histological examination was carried out as part of this study to confirm it. Increased mechanical sensitivity associated with neuroma development has been reported in humans undergoing both traumatic and elective digital amputations^37^.

While the effects of tail amputation on the central nervous system have not been directly examined in this study, reports from human and rodent studies suggests that peripheral nerve injury induces hyperexcitability changes that extend from the periphery, spinal cord and brainstem to several cortical structures^16^. Long-term synaptic depression in the insular cortex has been previously characterised in mice as a consequence of tail amputation^38^, supporting the hypothesis of central sensitisation following a peripheral injury. In order to confirm whether peripheral mechanical sensitisation is a valid proxy measure for the experience of pain in pigs, more comprehensive experimental approaches would be required, coupling quantitative sensory testing with more complex behavioural non-reflexive assays (e.g. motivational tasks) that provide more information on the involvement of the central nervous system in the affective component of pain experienced by pigs following tail amputation.

Age at the time of tail amputation appears to have influenced the temporal changes in MNTs. Significantly lower values were recorded 16 weeks post-tail resection in pigs exposed to surgery at 9 weeks of age when compared to MNTs of intact tails. In pigs undergoing tail resection at 17 weeks of age, MNTs were significantly lower 8 but not 16 weeks post-surgery.

Previous studies on surgical neuropathic pain models in both young and aged rats have demonstrated the occurrence of tactile allodynia up to 35 days following surgery^39, 40^. In both studies the effect of age at the time of injury was evaluated and a lower magnitude of change in allodynia, with less robust (i.e. lower withdrawal response thresholds) neuropathic pain behaviours, was observed in older rats. Age-specific differences in the duration of sensitisation may reflect different stages of maturation of the peripheral and central components of the nervous system at the time of the insult. In humans and rodents, the level of maturity of the nervous system has been suggested as a determining factor in the responsiveness to tactile and noxious stimulation^41^. Younger subjects appear to exhibit more pronounced and prolonged behavioural and electrophysiological responses to noxious stimulation, which decreased in duration over time^42, 43^. It is possible that tail resection had a different impact on long-lasting changes in nociceptive neural pathways according to the neuroanatomical maturity of the animals at the time of surgery although, based on the small sample size of pigs (8 animals per treatment) tested at 16 weeks following surgery at 17 weeks of age, interpretation of the temporal pattern of sensitisation should be made with some degree of caution. Nine and seventeen week-old pigs are at distinct stages of growth (i.e. early juvenile and pubertal late juvenile) and have different degrees of brain and spinal cord development^44–46^, which may influence somatosensory and affective responses to amputation injury. In addition, it should be recognised that another factor that can determine the intensity and duration of post-amputation sensitivity is the different degree of hyper-innervation by A- and C-fibres around the injured area during the healing process^41^. Sprouting of sensory nerves after traumatic injury is a known phenomenon in humans and rodents and is particularly marked in young subjects (neonates) and linked to peripheral hypersensitivity and central sensitisation^47^. It is not known if such hyper-innervation of healed tissues after amputation injury is associated with sustained peripheral sensitivity in juvenile pigs.

Another explanation for the apparent lack of a sustained effect on MNT observed between intact and tail-resected pigs 4 month after surgery at 17 weeks of age may be an effect of age on the rate of peripheral axonal regeneration after amputation injury. Age-related changes in the time and extent of post-operative tissue re-innervation (density and number of regenerating axons) is slower and decreased in older animals^48^.

The amount of tail removed following amputation (1/3^rd^ “long” vs. 2/3^rds^ “short”) had different effects on MNTs depending on the age at which tail amputation was performed and the sampling time after tail resection. No tail length treatment effect was observed one week following tail amputation in either age group. Similarly, short and long tails exhibited similar MNTs in later testing of pigs undergoing surgery at 9 weeks of age. In contrast, a later tail length effect was observed in pigs exposed to surgery at 17 weeks of age, with significantly lower MNTs in the long tail group 2 months after tail amputation compared to the short tail pigs, although it was not possible to evaluate the effect of tail amputation length 4 months after surgery owing to a mechanical breakdown of the PAM device which prevented the assessment and collection of data from the long tail resected pigs at this time point. The finding that pigs exposed to a greater extent of tail amputation appeared less sensitive to mechanical stimuli may contradict previous human literature. It is recognised that the greater the level of amputation injury in limbs and appendages in humans the greater the potential for the presence and severity of pain over a larger affected area due to proportionally greater damage occurring to somatosensory peripheral nerves^49^. Nonetheless, the emergence of phantom pain appears independent of the level of amputation^50^. The observed reduction in nociceptive thresholds measured in the current study may be explained by gradients in somatosensory innervation in different regions of the tail^27^. Proximal-distal variations in intra-epidermal nerve fibre innervation densities have been reported in human studies with greater densities recorded in the most distal parts of the leg^51^. Confirmation of a similar pattern of innervation density has yet to be corroborated in the pig tail. In addition to possible regional changes in tail somatosensory nerve distribution, it is possible that reductions in MNT in amputated tail stumps of differing lengths may be affected by the relative thickness of the soft-tissues at the site of mechanical stimulation. In humans, distinct values of nociceptive thresholds are linked to the varying thickness of cutaneous, adipose and muscle tissues^52, 53^. Lower MNT observed in the long tails may therefore reflect greater and more rapid focal noxious mechanical force generation linked to the reduced thickness of the overlying soft-tissues in the most distal part of the amputated tail. The converse may be true where the tissues at the site of stimulation are thicker, which may dissipate some of the mechanical force before response thresholds are achieved.

In conclusion, tail amputation injury in pigs appears to evoke acute as well as sustained changes in peripheral mechanical sensitivity with hyperalgesia observed one week and up to several weeks following the surgical procedure. The age at which the animals are exposed to the injury seemingly influences the temporal profile of sensitisation, with younger pigs being affected for a longer period of time. Further studies on age and body size-specific morphological characteristics are needed to fully comprehend the relationship with post-injury pain sensitivity in pigs. In addition, a smaller extent of tail amputated appears to be associated with higher sensitisation; however the partial loss of data collected in older animals warrants particular caution in interpreting our findings. In the near future, it will be of pivotal importance to correlate these mechanical threshold measures of pain sensitivity with changes in molecular mediators of pain signalling to provide novel information on the mechanisms and pathways involved in the development of hyperalgesia in pigs following tail amputation.

## Methods

### Animals and housing

A total of 108 female pigs, *Sus scrofa domesticus* (Landrace/Large White X synthetic sire line, Hermitage Seaborough Ltd., North Tawton, UK) from the resident herd at Cockle Park Farm, Newcastle University, were used in this study. Pigs of two distinct ages were used: (1) age of 9 weeks and a mean body weight of 22.3 ± 3.1 kg (n = 41); (2) age of 17 weeks and a mean body weight of 58.5 ± 8.4 kg (n = 67). For the purpose of the study, all animals retained intact tails until the time of tail resection and were ear-tagged for identification within the first week of their life. Animals identified with any tail biting or body injuries at any time prior to surgical tail resection were excluded from the study. From selection at the time of farrowing until one week prior to the beginning of the study, the pigs were maintained under standard commercial conditions, with *ad libitum* access to feed and water.

### Experimental design

In order to examine the interaction between age at the time of surgery and post-amputation tail pain sensitivity, mechanical nociceptive thresholds (MNT) were measured on the tails of pigs undergoing surgical tail resections at 9 and 17 weeks of age. All pigs exposed to surgery at 9 weeks of age were tested for MNT three days pre-and one week post-surgery (n = 41). A sub-sample of this group was tested again 16 weeks after surgical tail resection (n = 19). Pigs undergoing surgery at 17 weeks of age were tested for MNT three days pre- and one week post-surgery (n = 67). A sub-sample of this group was tested for tail MNT again either at 8 (n = 24) or 16 weeks following surgical tail resection (n = 14) (Table 1). These subsets contained a reduced number of animals due to euthanasia of groups for molecular study of tissues at different stages post resection.

**Table 1:** Overview of the number of animals from which mechanical nociceptive threshold (MNT) data were collected at each individual time point (number of days/weeks in relation to the time of tail resection) (^†^data missing due to behavioural non-compliance of animals; *data missing due to technical failure of Pressure Application Measurement device).

Age at surgery (weeks)	Tail treatment	MNT testing time point			
		*3 days pre*	*1 week post*	*8 weeks post*	*16 weeks post*
*9*	*Intact*	13	13	—^†^	6
	*Short*	16	16	—	8
	*Long*	12	12	—	5
*17*	*Intact*	23	23	8	6
	*Short*	24	24	8	8
	*Long*	20	20	8	—*

To assess the effect of the extent of tail resected on MNT, pigs were randomly assigned to one of three experimental treatments: (1) intact – pigs retained original tail lengths; (2) short tail – two-thirds of the intact tail length removed; (3) long tail – one-third of the intact tail length removed. Tail lengths were based on recent findings suggesting an effect of the extent of tail resected on the prevalence of neuromas and on neonatal behaviours^20, 54^.

### Anaesthesia, surgery and recovery

Food was withheld for a minimum of eight hours before surgery. On the day of surgery, all pigs housed in one pen were transferred to a building comprising temporary holding pens and a surgical theatre. Prior to surgery, individual animals were weighed and received an intramuscular injection of ketamine (5 mg/kg, Vetoquinol, Buckingham, UK), midazolam (0.5 mg/kg, Hameln, Gloucester, UK) and medetomidine (10 μg/kg, Vetoquinol, Buckingham, UK). Following injection, the animals were left undisturbed in an individual pen while being monitored by a trained animal research technician, and transferred to the surgical theatre once sedation was achieved (i.e. loss of righting reflex). Each pig was placed in lateral recumbence on the operating table and anaesthesia was maintained with the use of isoflurane (3%–4%) (Abbot Laboratories Ltd, Queenborough, Kent, United Kingdom) delivered in 100% oxygen. During anaesthesia, blood O_2_ saturation (Nonin Plymouth, MN, USA) and body temperature (Sigma Aldrich, Gillingham, UK) were continually monitored. The tail length was measured to determine the location for resection based on the pre-assigned randomly-determined treatment. The tail was thoroughly cleaned and swabbed with 7.5% antiseptic povidone-iodine solution (Ecolab, Leeds, UK) and a tourniquet was placed at the base of the tail to reduce blood flow. Loss of tail reflex was verified before initiating the resection, which was performed with Liston bone cutting forceps (World Precision Instrument, Hertfordshire, UK). Pinpoint haemostasis was performed with a high temperature cautery pen (Bovie Medical Corporation, Clearwater, FL, USA) and wound powder was applied to the distal tail stump. Once tail resection was completed, anaesthesia was interrupted by discontinuing isoflurane and providing 100% oxygen until recovery of righting reflex. In addition, the pigs received an intramuscular injection into the neck muscle of atipamezole (5000 mcg/m^2^, Vetoquinol, Buckingham, UK), meloxicam (0.2 mg/kg, Boehringer Ingelheim, Bracknell, UK) and Penicillin-Streptomycin (Norbrook, Corby, UK). At the end of the procedure, each pig was covered with a thermal blanket and transferred to an individual recovery pen measuring 2 × 2 m and containing deep clean straw as bedding material and a water drinker. For the following two days, pigs were housed individually in adjacent recovery pens that allowed visual, auditory and olfactory cues but prevented physical interaction with other animals. They received feed ad libitum and daily intramuscular injections of penicillin-streptomycin (Norbrook, Corby, United Kingdom) to prevent infections. The animals designated to the ‘intact’ treatment were exposed to the same protocol, except for the amputation of a portion of the tail and the administration of post-surgery drugs (i.e. meloxicam, Penicillin-Streptomycin). The health of the animals was continually monitored during recovery by a trained operator along with regular inspections by the project veterinarian.

### Experimental set-up

Pigs of 17 weeks of age were tested inside a custom-made crate situated in a test room adjacent to the home pens. Briefly, the crate consisted of steel mesh side panels (1.8 m x 1 m) with a front and a rear gate, to allow the animals to consistently enter at one end and leave from the other. The crate set-up kept the pigs facing forward and prevented them from turning around. The crate incorporated a drinker (5 L volume), fixed to the front gate, which contained a 5% sucrose solution. In contrast, pigs of 9 weeks of age appeared to be too stressed by confinement in a crate, therefore testing of these animals occurred in a 3 × 3 m arena located in a room adjacent to the home pens. The arena consisted of a barren environment (concrete floor) with a feeder provided to the animals and walls made of 1 m high PVC boards. For both test scenarios a laptop computer was placed adjacent to the test area in a location that allowed the experimenter to observe screen while concurrently applying the stimuli. Full details of the experimental set-up have been previously described by Di Giminiani *et al*.^27^.

### Mechanical nociceptive threshold testing

Tail MNTs were measured as previously described by Di Giminiani *et al*.^27^. Briefly, a handheld digital Pressure Application Measurement (PAM) device (Ugo Basile, Varese, Italy) was applied to the dorsal surface of the tail at a rate of 120 g force per second (gF/s) until a withdrawal response was induced (tail flick or tail clamp) or when the maximal cut-off force of 1500 gF was reached. Post-surgical stimuli were applied in a site immediately adjacent to the injured section of the tail, at a maximum distance of one cm. Force application was monitored on a computer screen through dedicated software (DCA, Ugo Basile, Varese Italy) and allowed the operator to provide a stable stimulation by matching the actual rate against a pre-set visualised value. All stimuli were delivered with a minimum of 2 min inter-stimulus intervals.

The PAM device was operated by one investigator (PDG) throughout the study. Blinding to treatment was not possible due to the constraints of the study.

### Habituation and testing protocol

The habituation and testing protocols and set-ups correspond to those described by Di Giminiani *et al*.^27^. Prior to testing, the pigs were habituated to the experimental test conditions once daily for four consecutive days, during which time they were allowed to walk freely in randomly selected pairs of pen-mates to the test set-up (i.e. arena or crate). Duration of confinement of the pigs increased gradually on a daily basis (2-min increase/day) reaching a maximum of 13 min on day four. Throughout habituation and testing, two observers followed the animals as they walked from the home pen to the test room and back and were present during the time of confinement. For the entire duration of the habituation period, 9 week-old pigs received 60% of the daily feed ration while in the test arena. Older animals assessed in the test crates had access to sucrose solution through the drinkers.

Each test session consisted of a total of nine mechanical stimuli applied in triplicate to each of three tail regions (proximal, medial, distal), as described in Di Giminiani *et al*.^27^. Following the reduction in tail length induced by the surgical resection, post-op MNTs were recorded in anatomical locations along the tail that corresponded to the pre-surgery stimulation sites. At the time of testing, the pigs followed the same procedure as during the habituation phase and the delivery of mechanical stimuli commenced as soon as the pigs started feeding (arena) or drinking the sucrose solution (crate). In order to accurately and consistently deliver the mechanical stimuli, the tail was manually placed inside a half-section of PVC tube (length: 20 cm; diameter: 4 cm) held by the operator. Gentle downward force was applied to the dorsal surface of the tail by hand to fully extent the tail inside the retaining tube. Application of the noxious mechanical stimulus elicited two highly consistent withdrawal responses: (1) a tail flick, defined as a rapid horizontal movement of the tail retracting away from the stimulus, and (2) a tail clamp, which consisted of muscle contraction-related downward pressure produced by the tail within the tube. In the absence of a response by the animal within the maximal cut-off value of 1500 gF, the measurement was recorded as 1500 gF (censored data) but was excluded from the analysis. Once all tail MNT measurements were obtained, the animals were released from test confinement and returned to their home pen. Typically, a test session, comprising the time required for the pigs to reach the test room, be tested and returned to the home pens, lasted on average 18 min (range: 15–20 min).

### Statistical analysis

In order to establish whether the age at the time of tail resection and the extent of tail amputation influenced the degree of variability in MNTs, the coefficient of variation (CV) was calculated for each animal at each available sampling time point and was analysed using a two-way repeated measures ANOVA with age at surgery and tail treatment (‘intact’, ‘short’ and ‘long tail’) as fixed factors. In order to assess the changes in MNT one week following sham/tail amputation surgery, a one-way repeated measures ANOVA was performed with tail-resection treatment as fixed factor, which included three levels (‘intact’, ‘short’ and ‘long tail’). Data were analysed using SPSS version 22.0 for Windows (SPSS Inc., Chicago, IL, USA).

Differences in MNTs across time and treatment groups were analysed with a linear mixed model (PROC MIXED) with age at the time of surgery (9 and 17 weeks), time of testing (1, 8 and 16 weeks post-surgery) and tail-resection treatment (‘intact’, ‘short’ and ‘long tail’) as fixed factors. The model included ‘pig’ as a random effect and baseline values of MNTs as covariate. A compound symmetry correlation structure was used to account for the clustering of repeated measurements within a session. Data were analysed with SAS 9.4 (SAS Institute Inc., Cary, NC, USA). All data are presented as means ± SEM with significance at P < 0.05.

### Ethical statement

All animal procedures were performed in accordance with the International Association for the Study of Pain guidelines for the Use of Animals in Research and carried out under licence (PPL 70/7919) granted under the Animal (Scientific Procedures) Act 1986 and approved by the Animal Welfare and Ethical Review Body of Newcastle University.
